# Economic evaluation of carbetocin as prophylaxis for postpartum hemorrhage in the Philippines

**DOI:** 10.1186/s12913-020-05834-x

**Published:** 2020-10-26

**Authors:** Jamaica Roanne Briones, Pattarawalai Talungchit, Montarat Thavorncharoensap, Usa Chaikledkaew

**Affiliations:** 1 Mahidol University Health Technology Assessment (MUHTA) Graduate Program, Mahidol University, Bangkok, Thailand; 2 Department of Obstetrics and Gynecology, Faculty of Medicine Siriraj Hospital, Mahidol University, Bangkok, Thailand; 3 Social and Administrative Pharmacy Division, Department of Pharmacy, Faculty of Pharmacy, Mahidol University, 447 Sri-Ayudhaya Rd., Phayathai, Ratchathewi, Bangkok, 10400 Thailand

**Keywords:** Carbetocin, Oxytocin, Postpartum hemorrhage, Economic evaluation, Philippines

## Abstract

**Background:**

The World Health Organization (WHO) recommends oxytocin as the drug of choice for postpartum hemorrhage (PPH) prevention. However, the WHO has also recently considered carbetocin for PPH prevention, but only if carbetocin were a cost-effective choice in the country. Consequently, we determined the cost-effectiveness and budgetary impact of carbetocin against oxytocin in the Philippines.

**Methods:**

A cost-utility analysis using a decision tree was done to compare the costs and outcomes of carbetocin with oxytocin for PPH prophylaxis among women undergoing either vaginal delivery (VD) or cesarean section (CS) in a six-week time horizon using a societal perspective. One-way and probabilistic sensitivity analyses were applied to investigate parameter uncertainties. Additionally, budget impact analysis was conducted using a governmental perspective. Results were presented as incremental cost-effectiveness ratio (ICER) using a 2895 United States dollar (USD) per quality adjusted life year (QALY) gained as the ceiling threshold in the Philippines.

**Results:**

Carbetocin was not cost-effective given the listed price of carbetocin at 18 USD. Given a societal perspective, the ICER values of 13,187 USD and over 40,000 USD per QALY gained were derived for CS and VD, respectively. Moreover, the ICER values were sensitive to the risk ratio of carbetocin versus oxytocin and carbetocin price. On budget impact, the five-year total budget impact of a drug mix of carbetocin and oxytocin was 25.54 million USD (4.23 million USD for CS and 21.31 million USD for VD) compared with ‘*only oxytocin’* scenario.

**Conclusion:**

Carbetocin is not a cost-effective choice in PPH prevention for both modes of delivery in the Philippines, unless price reduction is made. Our findings can be used for evidence-informed policies to guide coverage decisions on carbetocin not only in the Philippines but also in other low and middle-income countries.

**Supplementary Information:**

The online version contains supplementary material available at 10.1186/s12913-020-05834-x.

## Background

Postpartum hemorrhage (PPH) remains as the leading cause of maternal morbidity and mortality worldwide. It accounts for 13% maternal deaths in developed countries and 28% of maternal deaths in developing countries, further disproportionately affecting those in the world’s poorest countries [1, 2]. While avoidable, the Philippines is still among the countries with high maternal deaths. In 2015, 114 deaths per 100,000 live births were registered in the country – a far cry from the target of 52 deaths per 100,000 live births [3]. Of these deaths, 30% were reportedly associated with PPH.

PPH is defined as a blood loss of more than 500 mL in vaginal delivery (VD) or more than 1000 mL in cesarean section (CS) and may lead to sequelae such as massive blood transfusion, ICU admission, or in extreme cases, hysterectomy [4–7]. It is unpredictable as it occurs without identifiable clinical or historical risk factors. Hence, effective prevention strategies such as the active management of the third stage of labor (AMTSL) are advocated for routine clinical practice [8]. AMTSL has three components: (1) administration of prophylactic uterotonic, (2) controlled cord traction, and (3) uterine massage, where emphasis is given to the use of a prophylactic uterotonic drug as it reduces the risk of PPH by 66% compared with the other two components [9–11].

Ideally, a prophylactic uterotonic should be given to all women on the third stage of labor [11, 12]. Several uterotonics are available for this purpose such as oxytocin, ergometrine, misoprostol, and more recently, carbetocin. Among these, the World Health Organization (WHO) recommends one dose of oxytocin (10 IU) as the drug of choice for the prevention of PPH and is also the drug of choice for PPH prevention in the Philippines [12, 13]. However, the WHO has also recently considered carbetocin as a prophylactic uterotonic of choice, but only if carbetocin were a cost-effective choice in the country [12].

Carbetocin is a synthetic analogue of oxytocin, which serves as an alternative to oxytocin. From the meta-analysis performed by Gallos and colleagues, carbetocin illustrated a longer duration of action compared with oxytocin, reducing the need for additional doses [risk ratio, RR (95% CI): 0.48 (0.34–0.68)]. It was also clinically effective in PPH prevention in VD [PPH ≥ 500 mL, RR (95% CI): 0.67 (0.34–1.30)] or in CS [PPH ≥ 1000 mL, RR (95% CI): 0.62 (0.31–1.23)] with comparable side effect profile to oxytocin [2]. Considering that oxytocin is not available in heat-stable preparations, the new formulation of carbetocin which can remain stable at 30 °C for 36 months would be widely appealing particularly in countries where cold chain would be a challenge such as in the Philippines [13, 14].

A systematic review indicated that seven out of eight cost-effectiveness studies involving carbetocin and oxytocin in high-, upper-middle, or middle-income countries concluded that carbetocin was cost-effective for CS, but no similar studies in low and middle-income countries (LMIC) were identified [15]. This raises the question of whether carbetocin would represent good value for money in an LMIC like the Philippines, especially that the price of carbetocin (13.10–25.60 USD) is much more expensive compared with oxytocin (0.27 USD) [15–17].

Late 2019, carbetocin was included in the Philippine National Formulary (PNF), the reference reimbursement list for essential medicines in the country. The inclusion was based on the non-inferior efficacy and safety profile of carbetocin compared with oxytocin, in which the cost-effectiveness of carbetocin was not considered before its inclusion given the lack of such evidence [18]. To fill the gap in evidence, we aimed to evaluate the cost-utility and budget impact of carbetocin compared with oxytocin for PPH prophylaxis among women giving birth through VD and CS in public hospitals. The results of this study can be used as a reference for evidence-informed policy decision on whether carbetocin should be still included in the PNF, and can also guide coverage decisions on carbetocin in other LMICs.

## Methods

A cost-utility analysis using a decision tree model was performed to assess the cost and health outcomes of one dose of carbetocin (100 μg) compared with oxytocin (10 IU), in women giving birth through VD and CS in a public hospital setting. Results were presented as an incremental cost-effectiveness ratio (ICER) in terms of United States dollar (USD) per quality adjusted life year (QALY) gained. A cost-effectiveness threshold of one annual gross domestic product (GDP) per capita or 2895 USD per QALY gained was applied, since the willingness-to-pay per QALY gained is not yet determined in the Philippines [19].

Cost-utility analysis was conducted based on a societal perspective in which direct medical, direct non-medical, and indirect costs were incorporated. All Philippine peso costs were converted to USD using the average exchange rate for the year 2019 (1 USD = 51.80 PHP) [20], while health outcomes were presented as QALY gained. Moreover, both costs and health effects were evaluated in six weeks; the postpartum period where all physiologic changes from pregnancy state return to a normal state (e.g., size of the uterus and hemodynamic changes), and considered to recover based on usual practice [21]. This period also incorporates the length of hospital stay if the patient would have a PPH complication for four weeks and recovery time for two weeks. Since outcomes were evaluated for six weeks, a discount rate was not applied.

Additionally, the budget impact of including carbetocin in the treatment mix for PPH prophylaxis compared with ‘*only oxytocin’* scenario was also evaluated for fiscal years 2020–2024 based on the governmental perspective (i.e., costs incurred by public hospitals and the fee-for-service covered by the PhilHealth, the government insurer).

### Model structure

**Fig. 1:**
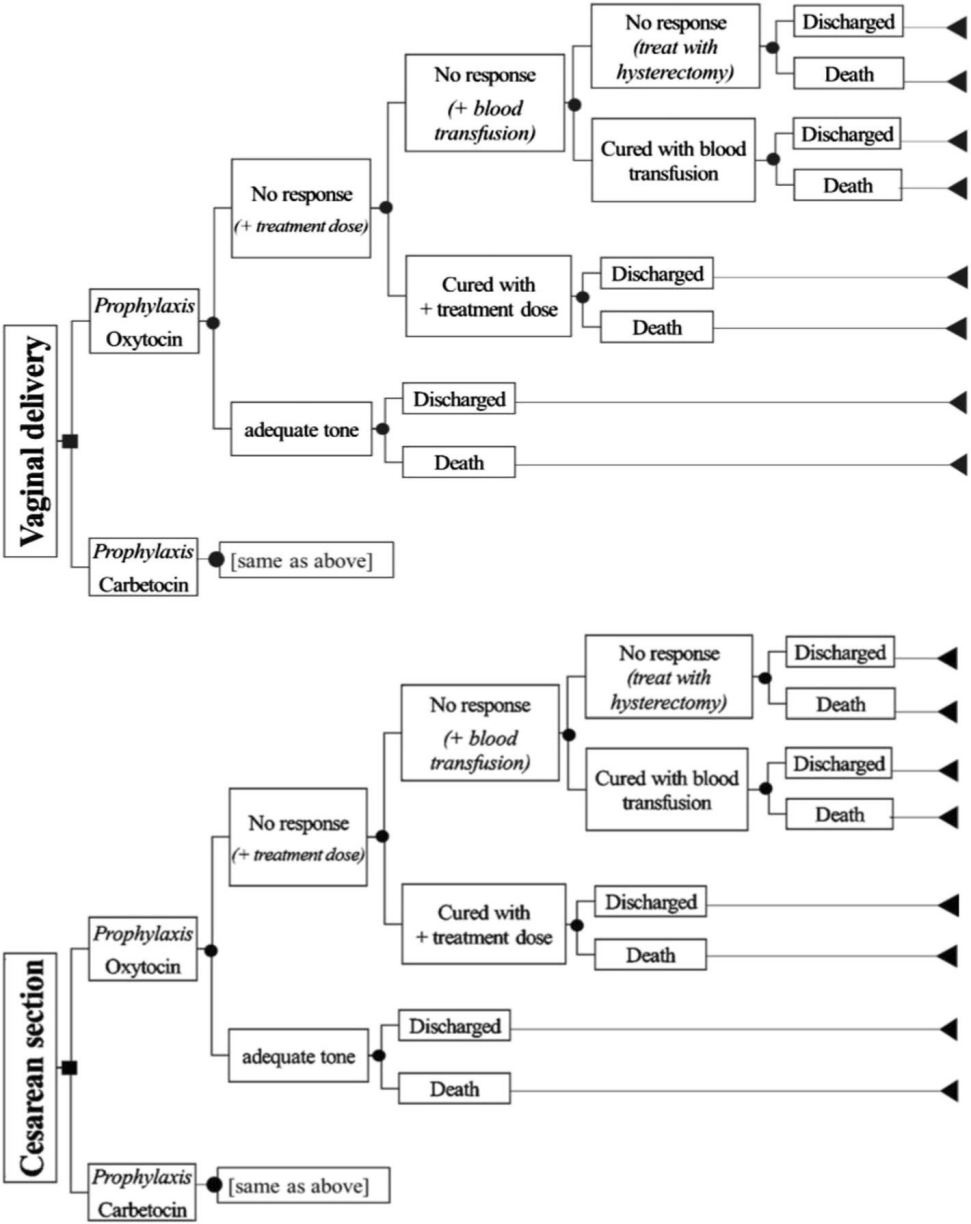
Decision tree model for the cost-utility analysis

The decision tree model was developed based on previous economic evaluations [9, 22–26], review of clinical practice guidelines [12, 27], and consultations with clinical experts in the Philippines (Fig. 1). We also based the model on the clinical pathway and treatment of PPH in the Philippines [28]. Although there are several factors related to PPH, we only considered interventions related to uterine atony. Further, we considered immediate PPH or event that occurred within 24 h of delivery in this study.

Two groups (i.e., women in VD or CS) were the study population. The model starts with one woman in either VD or CS who would receive a prophylactic dose of either oxytocin or carbetocin. After the initial dose, the patient who responds to the treatment may be discharged or may die. If she still experienced uterine atony, she would require additional uterotonic treatment of two to five doses. If bleeding was still not managed, a blood transfusion may be given. In rare and extreme cases, a patient may still not respond and a hysterectomy would be performed.

### Treatment effects

We obtained the treatment effects i.e., the RRs of carbetocin compared with oxytocin from the network meta-analysis done by Gallos and colleagues [2] for the clinical efficacy. The RRs of subgroup analysis on modes of delivery in preventing blood loss of more than 500 mL for VD, and more than 1000 mL for CS were used, following the definition of PPH for blood loss. Then, we multiplied the RRs to the baseline probabilities of corresponding nodes in the carbetocin arm i.e., no response nodes for ‘*plus treatment dose*’, ‘*plus blood transfusion*’, ‘*treat with hysterectomy*’, and all other death nodes (Fig. 1). Probabilities for ‘*plus treatment dose*’ and ‘*plus blood transfusion*’ were derived from the same meta-analysis using absolute probabilities relative to oxytocin. Incidence of *‘treat with hysterectomy*’ and maternal deaths were taken from a global survey conducted by the WHO using Philippine country data from seven health facilities [29]. Moreover, the probability values of ‘*treat with hysterectomy*’ and maternal deaths were assumed the same for women in VD and CS, since data were not disaggregated for the modes of delivery (Tables 1 and 2).

**Table 1:** Input values used in the analysis for cesarean section

Parameters	Values	Standard error	Distribution	Source
***Probability parameters***				
Probability of needing treatment dose with oxytocin, CS	0.2318	0.0119	beta	Meta-analysis [2]
Probability of needing blood transfusion with oxytocin, CS	0.0287	0.0390	beta	Meta-analysis [2]
Probability of needing hysterectomy with oxytocin, CS	0.00134	0.0003	beta	Global survey [29]
Probability of maternal deaths with oxytocin, CS	0.00134	0.0003	beta	Global survey [29]
Risk ratio of carbetocin as prophylaxis in CS (PPH ≥ 1000 mL)	0.62	0.23	lognormal	Meta-analysis [2]
Probability of needing treatment dose with carbetocin, CS	0.1437	0.0119	beta	Meta-analysis [2]
Probability of needing blood transfusion with carbetocin, CS	0.0178	0.0390	beta	Meta-analysis [2]
Probability of needing hysterectomy with carbetocin, CS	0.00083	0.0003	beta	Global survey [29]
				Meta-analysis [2]
Probability of maternal deaths with carbetocin, CS	0.00083	0.0003	beta	Global survey [29]
				Meta-analysis [2]
***Cost parameters***				
*Direct medical costs*				
No complication (n = 31)	445.01	13.87	gamma	Primary data collection
Additional treatment dose (*n* = 31)	465.04	11.46	gamma	Primary data collection
Blood transfusion (n = 13)	584.99	22.98	gamma	Primary data collection
Hysterectomy (n = 12)	987.68	87.05	gamma	Primary data collection
*Direct non-medical costs*				
No complication	33.12	2.11	gamma	Primary data collection
Additional treatment dose	45.17	2.49	gamma	Primary data collection
Blood transfusion	54.57	8.32	gamma	Primary data collection
Hysterectomy	57.08	5.82	gamma	Primary data collection
*Indirect cost of one caregiver*				
No complication	50.79	2.28	gamma	Primary data collection
Additional treatment dose	67.24	3.93	gamma	Primary data collection
Blood transfusion	85.77	12.84	gamma	Primary data collection
Hysterectomy	57.08	5.82	gamma	Primary data collection
***Utility parameters***				
Utility in CS, no complications (n = 77)	0.83	0.02	beta	Primary data collection
Utility in CS, with PPH episode (n = 3)	0.67	0.15	beta	Primary data collection
Utility in CS, with hysterectomy	0.50	–		Visual analogue scale, expert advise [30]

**Table 2:** Input values used in the analysis for vaginal delivery

Parameters	Values	Standard error	Distribution	Source
*Probability parameters*				
Probability of needing treatment dose with oxytocin, VD	0.1067	0.0025	beta	Meta-analysis [2]
Probability of needing blood transfusion with oxytocin, VD	0.0134	0.0153	beta	Meta-analysis [2]
Probability of needing hysterectomy with oxytocin, VD	0.0013	0.0003	beta	Global survey [29]
Probability of maternal deaths with oxytocin, VD	0.0013	0.0003	beta	Global survey [29]
Risk ratio of carbetocin as prophylaxis in VD (PPH ≥ 500 mL)	0.67	0.12	lognormal	Meta-analysis [2]
Probability of needing treatment dose with carbetocin, VD	0.0715	0.0025	beta	Meta-analysis [2]
Probability of needing blood transfusion with carbetocin, VD	0.0091	0.0153	beta	Meta-analysis [2]
Probability of needing hysterectomy with carbetocin, VD	0.00089	0.0003	beta	Global survey [29]
				Meta-analysis [2]
Probability of maternal deaths with carbetocin, VD	0.00089	0.0003	beta	Global survey [29]
				Meta-analysis [2]
**Cost parameters**				
*Direct medical costs*				
No complication (n = 38)	180.09	6.15	gamma	Primary data collection
Additional treatment dose (*n* = 37)	185.54	10.35	gamma	Primary data collection
Blood transfusion (n = 13)	316.12	11.54	gamma	Primary data collection
Hysterectomy (n = 12)^a^	987.68	87.05	gamma	Primary data collection
*Direct non-medical costs*				
No complication	33.84	2.22	gamma	Primary data collection
Additional treatment dose	35.38	2.72	gamma	Primary data collection
Blood transfusion	48.55	5.25	gamma	Primary data collection
Hysterectomy	57.08	5.82	gamma	Primary data collection
*Indirect cost of one caregiver*				
No complication	58.88	4.41	gamma	Primary data collection
Additional treatment dose	59.27	5.09	gamma	Primary data collection
Blood transfusion	62.49	4.50	gamma	Primary data collection
Hysterectomy	57.08	5.82	gamma	Primary data collection
***Utility parameters***				
Utility in VD, no complications (n = 108)	0.85	0.01	beta	Primary data collection
Utility in VD, with PPH episode (n = 8)	0.78	0.03	beta	Primary data collection
Utility in VD, with hysterectomy	0.50	–		Visual analogue scale, expert advise [30]

*PPH* Postpartum hemorrhage, ^a^same with cost in CS with hysterectomy

### Cost inputs

Using the societal perspective, direct medical and non-medical costs, as well as indirect costs of a caregiver were considered in the analysis. Direct medical costs were derived from hospital billing records of Dr. Jose Fabella Memorial Hospital (DJFMH), a tertiary level birthing hospital in Manila, Philippines with approximately 15,000 deliveries annually. A total of 175 hospital-billing records were retrospectively reviewed to derive the average cost of services for patients who gave birth through VD and CS, who did or did not have PPH-related complications in 2018. This contained information on inpatient fees such as drug, medical supplies, laboratory, procedure and other miscellaneous costs, and lastly, professional fees and room charges. Direct medical cost data were collected from women without complication (*n* = 31 for CS, *n* = 38 for VD), with additional treatment dose (n = 31 for CS, *n* = 37 for VD), blood transfusion (*n* = 13 for CS, n = 13 for VD), and hysterectomy (*n* = 12 for CS). Costs were calculated as cost-at-charge and converted to values in 2019 using the Philippine Consumer Price Index [31].

The ethical approval was granted by the Ethics Committee of DJFMH before retrieving hospital charges (REC-2019-004 ver. 1). A patient identifier was developed to protect the confidentiality of the patient since hospital-billing records contained patient’s personal information. The average cost and the sample size for each health state evaluated are presented in Table 1 for CS and Table 2 for VD, while the supplementary file provides details of the sampling method (Table S1).

Since carbetocin was not available in the hospital formulary at the time of data collection, we could not retrieve other healthcare costs related to its use from hospital bills. To estimate healthcare costs associated with carbetocin use, we assumed a product switch of oxytocin to carbetocin by subtracting the price of one dose of oxytocin (0.21 USD [17, 32]) to the total inpatient cost and adding the price of one dose of carbetocin (18.01 USD [16]). Only one dose replacement was considered for all decision nodes since carbetocin is intended for single-use administration only [33]. Another data limitation was that only twelve patients underwent hysterectomy following childbirth delivery for the year 2018, with no hospital records of patients in VD who underwent hysterectomy found. With this, we assumed the same cost for hysterectomy regardless of the mode of delivery.

Direct non-medical costs included food and transportation costs for the patient and one caregiver. Food costs were estimated through a standardized per capita budget of 2.90 USD for inpatient meals per day in government hospitals multiplied by the length of stay identified in the hospital bills [34]. Transportation costs were estimated from the distance of the province of the patient to the hospital. This was also available in the hospital bills and we computed the cost per kilometer based on the public fare matrices [35]. For indirect costs of one caregiver, income lost due to missed days of work was estimated. The minimum wage per day was multiplied by the total days of absenteeism, estimated as the length of stay and one day of outpatient visit. The same direct non-medical and indirect costs were assumed for oxytocin and carbetocin use. Semi-structured interviews with key informants such as obstetricians, anesthesiologists, nurses and administrative officers were performed to validate the assumptions for direct non-medical cost and indirect cost calculation.

### Utility values

Utility values used in this study were obtained from primary data collection using the EQ-5D-5L questionnaire from March 13 to June 30, 2019 at Siriraj Hospital – the largest tertiary and quaternary-care hospital in Thailand [36]. The study was conducted after the ethical approval granted by the Institutional Review Board (IRB) of Siriraj Hospital (*COA. No. Si 128/2019*). Permission from the EuroQoL group was received before using the EQ-5D-5L questionnaire (*Registration ID: L-29103*). The inclusion criteria for the survey were: (1) Thai women aged 18 years old and over who were postpartum and received treatment in the postpartum ward, (2) those with normal vital signs and conscious, (3) those who could interact, make decisions on their own and communicate in Thai; (4) those who did not have a disease which affects the quality of life (e.g., psychiatric diseases – depression, movement disability, not able to do daily activities on her own). Patients were excluded if they were unable to complete the quality of life assessment form and if the case record form were incomplete. A total of 196 Thai women who gave birth through VD and CS were included based on the inclusion and exclusion criteria. Utility data were collected from women without complication (*n* = 77 for CS, *n* = 108 for VD) and those with PPH episode (*n* = 3 for CS, *n* = 8 for VD). The utility values and the sample size for each health state evaluated are presented in Table 1 for CS and Table 2 for VD, while the supplementary file contains details on sample size computation (Table S2).

Patients willing to join the research study were requested to sign consent forms and subsequently asked to answer the EQ-5D-5L questionnaire. Interviews to obtain the patients’ health utility values were conducted within three days postpartum while still admitted to the hospital. Data collectors assisted the patients if they could not understand, read, or answer the questionnaire. A patient identifier was developed to protect the identity of the patient.

The EQ-5D-5L survey results were converted to utility scores ranging between 0 (*worst quality of life*) to 1 (*best quality of life*) using the Thai EQ-5D-5L value set [37]. In assigning weights, patients needing ‘*prophylactic dose only*’ and ‘*plus treatment dose*’ were assigned with utilities of VD or CS without complications. Those who needed blood transfusion were assigned with utilities of VD or CS with PPH episodes. For utility weight associated with hysterectomy, we referred to a study in Israel where they determined utility values for patients who underwent CS following hysterectomy [30]. QALYs were then calculated by multiplying utility scores and the number of years that the patients were alive which were converted from the six-week time horizon used in the analysis.

### Uncertainty analysis

One-way sensitivity analysis was performed to determine which of the parameters would cause the greatest variation in ICER. The standard error of each parameter was applied to test parameter uncertainty. Where no standard errors for probabilities were provided in literature estimates, they were calculated as ±20% of the mean. For unreported 95% confidence intervals (CI), these were assumed as ±10% of the mean. Additionally, a threshold sensitivity analysis was performed to determine the price at which carbetocin would be cost-effective for VD and CS at the cost-effectiveness threshold of one annual GDP per capita or 2895 USD per QALY gained. We also explored the sensitivity of the results with a carbetocin price offer at 0.31 USD, which is the subsidized price that the manufacturer would offer to public healthcare facilities in LMIC once regulatory bodies approve the registration of carbetocin [38]. The effect of parameter uncertainties on ICER was also explored through probabilistic sensitivity analysis (PSA), using Monte Carlo simulation with 1000 iterations and illustrated through the cost-effectiveness planes and cost-effectiveness acceptability curves (CEAC) using Microsoft Excel 2017.

### Budget impact analysis

The budget impact of including carbetocin in the treatment mix for PPH prophylaxis compared with ‘*only oxytocin’* scenario was evaluated using a government’s perspective for fiscal years 2020–2024 using the same Excel-based model for cost-utility analysis. Patients who received prophylactic uterotonic was assumed as the total registered births in hospitals. The latest report for live births by the Philippine Statistics Authority was for 2017 with a total registered 1,700,618 live births. Since the number of registered live births declined by 5 % from 2012 to 2017, a 1 % decline from the previous year’s live births was assumed to calculate the eligible population [39]. Moreover, the proportion of patients attended by health professionals (93.3% for 2017) and the proportion of mode of delivery **(**19%, CS**)** was applied [40]. Given these assumptions, the number of patients likely to give birth in VD and CS were predicted for 2020–2024.

For cost inputs, we considered costs incurred by public hospitals and the fee-for-service covered by the PhilHealth, the entity that reimburses fee-for-services or case rates as commonly known in the country. Published case rates for 2017 were used as input values for costs [41]. Since there is no published data on product uptake of carbetocin, an initial product mix of 90% oxytocin and 10% carbetocin use was forecasted for the first year of use with a 5% increase in uptake of carbetocin for the succeeding years [42].

## Results

### Cost-effectiveness analysis

For women giving birth through CS, incremental cost and incremental QALY of carbetocin compared with oxytocin were 13.19 USD and 0.001 QALYs, resulting in the ICER value of 13,187 USD per QALY gained. For those in VD, incremental cost and incremental QALY of carbetocin were 17.49 USD and 0.000405 QALYs, leading to the ICER value of 43,164 USD per QALY gained compared with oxytocin. Regardless of the mode of delivery, the ICER values were over the cost-effectiveness threshold of one GDP per capita (2895 USD per QALY gained) which rendered carbetocin not cost-effective in the Philippines. Table 3 presents cost-effectiveness analysis results in reference to probabilistic ICER results, which were the average of 1000 iterations generated from the Monte Carlo simulation.

**Table 3:** Summary of cost-effectiveness analysis results

Probabilistic results	Carbetocin	Oxytocin
**Cesarean section (CS)**		
Total costs	555	542
Total QALYs	0.82797	0.82697
Incremental cost		13.19
Incremental QALY		0.001000
ICER per QALY gained (USD/QALY gained)		13,187
**Vaginal delivery (VD)**		
Total cost	292	274
Total QALY	0.84535	0.84494
Incremental cost		17.49
Incremental QALY		0.000405
ICER per QALY gained (USD/QALY gained)		43,164

We also estimated mortality reduction given that carbetocin was more effective in preventing PPH compared with oxytocin. Using the proportion of VD and CS births for the 2020 birth cohort, the estimated total maternal deaths in oxytocin arm were 394 in CS and 1668 in VD, while those in carbetocin arm were 244 in CS and 1117 in VD. From the projection, an estimate of 150 in CS and 550 in VD deaths can be averted if carbetocin was used instead of oxytocin.

### Uncertainty analysis

**Fig. 2:**
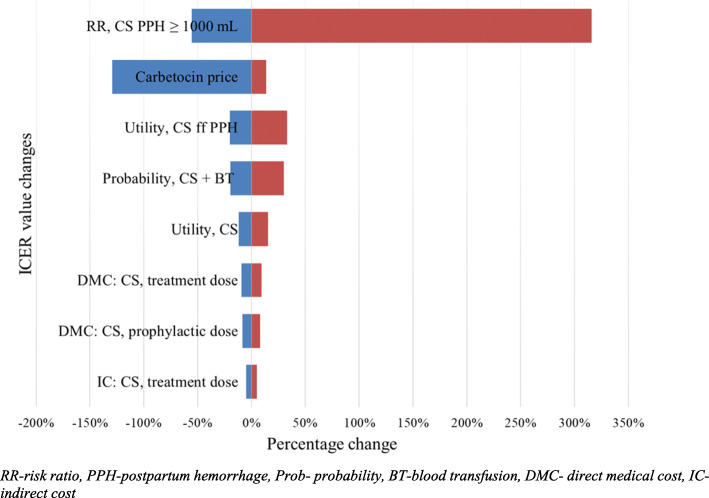
Tornado diagram for parameters used in CS analysis

ICER in CS was most sensitive to the risk ratio of carbetocin over oxytocin for PPH ≥1000 mL in CS delivery with a 370% change, followed by drug price (128%), utility in CS following PPH (53%), and probability in CS following blood transfusion (49%), illustrated in the tornado diagram (Fig. 2). The same parameters that influenced ICER values in VD were also observed to influence VD (Figure S1, Supplementary file). In VD, the risk ratio of carbetocin compared with oxytocin for PPH ≥ 500 mL had the most influence in ICER with over 250% change in range, followed by drug price (140%), utility in VD (23%) then cost of carbetocin (21%).

**Fig. 3:**
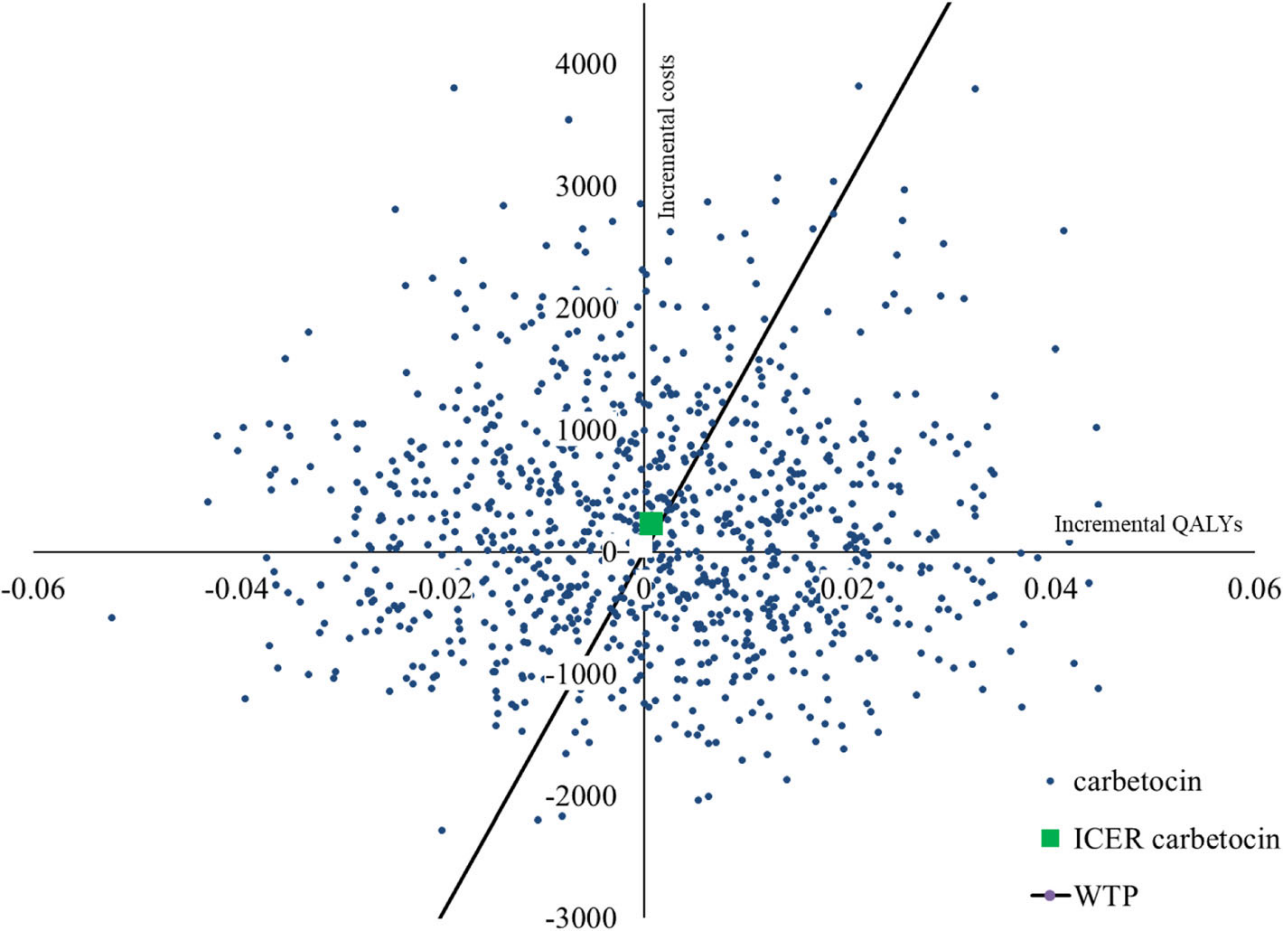
Cost-effectiveness plane of carbetocin compared with oxytocin for CS

**Fig. 4:**
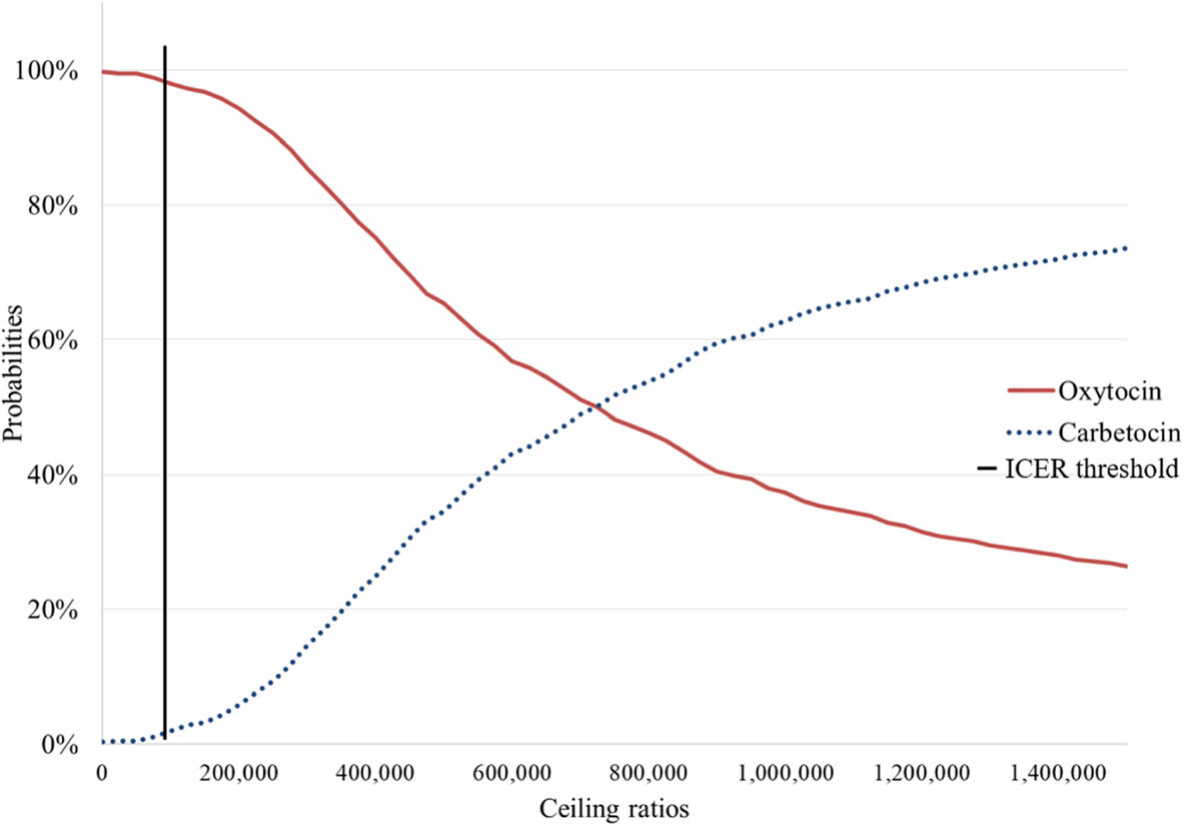
Cost-effectiveness analysis curve for CS analysis

The cost-effectiveness plane shows the incremental costs and incremental QALYs generated for carbetocin compared with oxytocin. Around 55% of the scenarios fell on the two quadrants on the right hand of the plane for CS (Fig. 3), and 49.5% for VD (Figure S2, Supplementary file). At one GDP per capita threshold, the probability of carbetocin to be cost-effective was only at 3% in CS (Fig. 4), while none of the scenarios was cost-effective in VD (Figure S3, Supplementary file). Carbetocin would only be cost-effective at the set threshold if the price were reduced by at least 90% (1.85 USD) for VD and 45% (8.26 USD) for CS, compared to the current price at 18.01 USD.

### Budget impact analysis

**Fig. 5:**
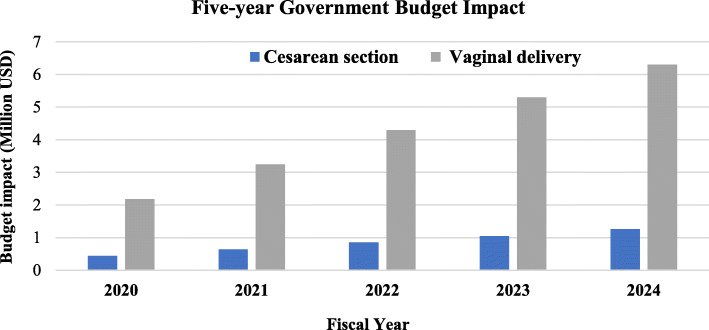
Budget impact for CS and VD with ‘drug mix- carbetocin and oxytocin’ and ‘only oxytocin’ scenario

The five-year budget impact of a drug mix of carbetocin and oxytocin compared with ‘*only oxytocin*’ scenario was computed for CS and VD (Fig. 5). With the assumed drug mix ratio, total drug costs would require a budget of 27.05 million USD for carbetocin use and 1.29 million USD in oxytocin for the entire birth cohort. When compared with ‘*only oxytocin’* scenario, the five-year total budget impact of a drug mix of carbetocin was 25.54 million USD (4.23 million USD for CS and 21.31 million USD for VD). Consequently, an increase in the percentage of carbetocin uptake would lead to a higher budget impact. The breakdown of cost components is in the Supplementary file, Tables S3 and S4.

## Discussion

In addition to oxytocin, the WHO also recommends carbetocin as a prophylactic uterotonic of choice, but only if it were a cost-effective choice in the country [12]. To the best of our knowledge, this study is the first to estimate the costs and health outcomes of carbetocin compared with oxytocin for PPH prophylaxis in the Philippines and an LMIC context. With the lack of evidence on cost-effectiveness of carbetocin before inclusion in the PNF [18], the results of this study can be used to guide coverage decisions whether carbetocin should still be listed. Additionally, we estimated the incremental budgetary requirement if carbetocin would be reimbursed.

Our study suggests that carbetocin is not cost-effective in both women giving birth through VD and CS delivery at one GDP per capita threshold in the Philippines compared with oxytocin. Our result is consistent with a published study in Colombia where carbetocin was not cost-effective in VD [23]. In contrast, compared to other published studies in a high-income country like the UK [43] and upper-middle-income countries such as Malaysia [44], Peru [22], Colombia [23] and Ecuador [24], carbetocin was consistently cost-effective for use in CS population, while our study reveals otherwise. The contrasting results can be attributed to the distinction in inpatient cost inputs among these countries compared to the Philippines. The cost inputs adopted in this study were quite low per inpatient day, as we derived from a government hospital that commonly caters to the most underprivileged population in the country. Philippine government facilities are mandated to implement the ‘No Balance Billing’ policy which subsidizes inpatient costs not covered by the fee-for-services reimbursed by PhilHealth if patients were eligible [45]. This was reflected in data we retrieved since out-of-pocket expenses for all the health states evaluated were only less than 5% of the total costs, mainly from patients who did not satisfy eligibility criteria for subsidy. Since the Philippines has a high proportion of private hospitals of around 60% [46], the costs derived from our sample would be entirely different if costs were also determined in a private facility. Additionally, the hospital charge in a public hospital is significantly lower compared to private health care facilities in the country. From a survey last 2007, average out-of-pocket expenditure among patients confined in private hospitals was 473 USD compared to 193 USD in public hospitals in the Philippines [46]. Nevertheless, since the aim of this study is to inform coverage decisions, using input for costs incurred in publicly managed and funded institutions is permissible.

Among the parameters used in the study, the risk ratio of carbetocin in outcomes leading to blood loss of ≥500 mL in VD and ≥ 1000 mL in CS was a major cause of uncertainty in our analysis. A reason for uncertainty may be due to the different methods of the pooled studies to quantify blood loss such as calibrated plastic drapes, visual estimation, or collection vessel at childbirth. The second reason could be that the different modes of administration of oxytocin (e.g., intravenous bolus plus an infusion of any dose or intravenous infusion only of any dose) were included. Albeit uncertainties, the point estimates used in the study are still reasonable guide on the relative treatment effects of carbetocin for these outcomes.

As shown in the results of the threshold sensitivity analysis on the price of carbetocin, a substantial decrease is necessary to render it cost-effective – from the current listed price at 18.01 USD to 8.26 USD for CS, and 1.85 USD for VD at the cost-effectiveness threshold of one GDP per capita in the Philippines. Although this cost-effective price is a drastic reduction, it may be possible since the manufacturer has offered the new heat-stable formulation of carbetocin at 0.31 USD to public healthcare facilities in LMICs [38]. This would favor carbetocin use regardless of the mode of delivery, as also reflected in the one-way sensitivity analysis results, where the price reduction reduced the ICER values at 128% and 140% for VD and CS, respectively.

Based on the forecasted financial impact, an increase in budget for drug cost along with a decrease in delivery costs was observed for the treatment mix scenario of oxytocin and carbetocin as compared with ‘*only oxytocin’* scenario. From the results, carbetocin use will be encouraging most especially if carbetocin price was lowered, as this could improve the affordability of the drug to government facilities. Moreover, since the results suggested that carbetocin required less budgetary requirements for CS than that for VD, its use for CS deliveries only can be considered.

It is important to address the limitations of this study. First, we reiterate the lack of actual hospitalization costs associated with carbetocin use, a parameter we had no choice but to estimate since it was just included in the PNF by 2019. Moreover, we considered the same cost of hysterectomy regardless of the mode of delivery. This was assumed since given a scenario of trial labor for VD, if the patient would go through severe bleeding and would need a hysterectomy, she would undergo CS eventually and classified as an emergency CS patient in the patient records. This justifies the assumption of similar cost in hysterectomy for VD or CS. The second data constraint is the absence of epidemiological data on some health states – the probability of maternal deaths in particular. The probability was assumed the same for both CS and VD for each of the chance nodes: (1) additional treatment dose to death (2) blood transfusion to death (3) hysterectomy to death and (4) adequate tone to death. This was due to data limitation on the probability of specific causes of maternal deaths and the probability of death on mode of delivery. Given that the health states involved in the model were very transient and acute as these usually happen within an hour during delivery, such details are not reported in existing literature. From the one-way sensitivity analysis, the probability of maternal deaths did not have much impact on the ICER values. We recognize this data limitation can overestimate the probability of death for women with adequate tone, but also underestimate the probability of death due to complications. Still, the probability values of maternal deaths between oxytocin and carbetocin arms were different in both CS and VD in differentiating effectiveness of the two drugs. Third, due to the lack of local utility values in the Philippines, we obtained the utility values from Thai population. Since Thai utility data are the only available and accessible data on an Asian population, this would be the most appropriate reference to our setting. We recommend that future studies should refer to local utility data. Lastly, the side effects were not considered in this study since carbetocin and oxytocin has the same side effects such as vomiting, fever and hypertension, hence may not have a differential impact on healthcare costs and health outcomes.

## Conclusions

Overall, the results of this study suggest that carbetocin is not a cost-effective choice in PPH prevention for both modes of delivery in the Philippines unless price reduction is made. Our findings can be used for evidence-informed policies to guide coverage decisions on carbetocin not just in the Philippines but also in resource-constraint settings such as LMICs with a similar policy question. On budget impact, compared to ‘*only oxytocin*’ scenario, the five-year total budget impact of a drug mix of carbetocin was 25.54 million USD (4.23 million USD for CS and 21.31 million USD for VD). Nonetheless, cost-effectiveness is beyond efficiency concerns. Given a possible intervention to address maternal morbidities, further work must be done. It is highly desirable to lower the price of carbetocin for use in a publicly-funded healthcare system, particularly in an LMIC where the drug would have a greater impact. Limiting use in cases such as patients with risk factors for PPH such as age and emergency CS patients may also be sensible as they may gain the most from the intervention. Furthermore, effective treatment is not always available in all settings, particularly when there are delays in referral such as in a community hospital setting where prophylactic regimens would be useful [47]. These scenarios were not analyzed given the limited available literature. Once data is available, future research could adopt this model using parameters in their respective settings. Although, one should note that prophylactic uterotonic is only one component of PPH prevention and would not work if other obstetric care components were not improved.

## Supplementary Information

**Additional file 1: Table S1.** Sample size for costing analysis. **Table S2.** Sample size for EQ-5D-5L survey **Figure S1.** Tornado diagram for parameters used in VD analysis. **Figure S2.** Cost-effectiveness plane of carbetocin compared with oxytocin for VD analysis. **Figure S3.** Cost-effectiveness acceptability curve for VD analysis. **Table S3.** Budget impact analysis results for cesarean section for 2020–2024 (*in millions*, USD). **Table S4.** Budget impact analysis results for vaginal delivery for 2020–2024 (*in millions*, USD).

## Data Availability

The cost and health utility data generated and analyzed during this study are available from the corresponding author on a reasonable request. Other parameters used in the economic model are cited accordingly.

## Abbreviations

AMTSL: Active management of the third stage of labor
CPI: Consumer price index
CS: cesarean section
CUA: Cost-utility analysis
GDP: Gross domestic product
ICER: Incremental cost-effectiveness ratio
LMIC: Low and middle income country
PNF: Philippine National Formulary
PSA: Probabilistic sensitivity analysis
QALY: Quality adjusted life year
VD: Vaginal delivery
WHO: World Health Organization
