# Supplementary material for: Economic evaluation of carbetocin as prophylaxis for postpartum hemorrhage in the Philippines
Source: BMC Health Serv Res. 2020 Oct 26;20:975. doi: 10.1186/s12913-020-05834-x (PMC7586682; doi:10.1186/s12913-020-05834-x)
Supplement: Supplementary file 1 — Additional file 1: Table S1. Sample size for costing analysis. Table S2. Sample size for EQ-5D-5L survey Figure S1. Tornado diagram for parameters used in VD analysis. Figure S2. Cost-effectiveness plane of carbetocin compared with oxytocin for VD analysis. Figure S3. Cost-effectiveness acceptability curve for VD analysis. Table S3. Budget impact analysis results for cesarean section for 2020–2024 (in millions, USD). Table S4. Budget impact analysis results for vaginal delivery for 2020–2024 (in millions, USD). [file 12913_2020_5834_MOESM1_ESM.docx]

**SUPPLEMENTARY FILE**

**Sampling method and sample size calculation**

Purposive sampling was done to retrieve hospital bill records for costing analysis and select patients for the EQ-5D-5L survey based on the inclusion and exclusion criteria for each of the studies. The sample size needed for each health state involved was calculated using the formula by Lemeshow et al^1^, where critical value (z) was set at 1.96 and standard error (Ɛ) at 0.1. The summary of the sample size calculated for costing analysis is in Table 1, while for EQ-5D-5L survey is in Table 2. References indicated are for values of mean and SD used in computing sample size.

Table S1. Sample size for costing analysis

| **Health states** | **Mean(PHP)** | **SD (PHP)** | **Sample size** | **Reference** |
| --- | --- | --- | --- | --- |
| Vaginal Delivery | 4,863 | 2,151 | 75 | ^2^ |
| Cesarean Section | 5,949 | 2,365 | 61 | ^2^ |
| Blood transfusion | 3,460 | 908 | 27 | ^4^ |
| Hysterectomy | 31,084 | 7,703 | 22 | ^3^ |

Table S2. Sample size for EQ-5D-5L survey

| **Health state** | **Mean (utility)** | **SD**  **(utility)** | **Sample size** | **Samples retrieved** | **Reference** |
| --- | --- | --- | --- | --- | --- |
| VD without complication | 0.57 | 0.3063 | 111 | 108 | ^5^ |
| CS without complication | 0.51 | 0.1730 | 44 | 77 | ^5^ |
| VD with PPH | 0.785 | 0.222 | 31 | 8 | ^6^ |
| CS with PPH | 0.785 | 0.222 | 31 | 3 | ^6^ |

**Uncertainty analyses graphs for vaginal delivery (VD)**


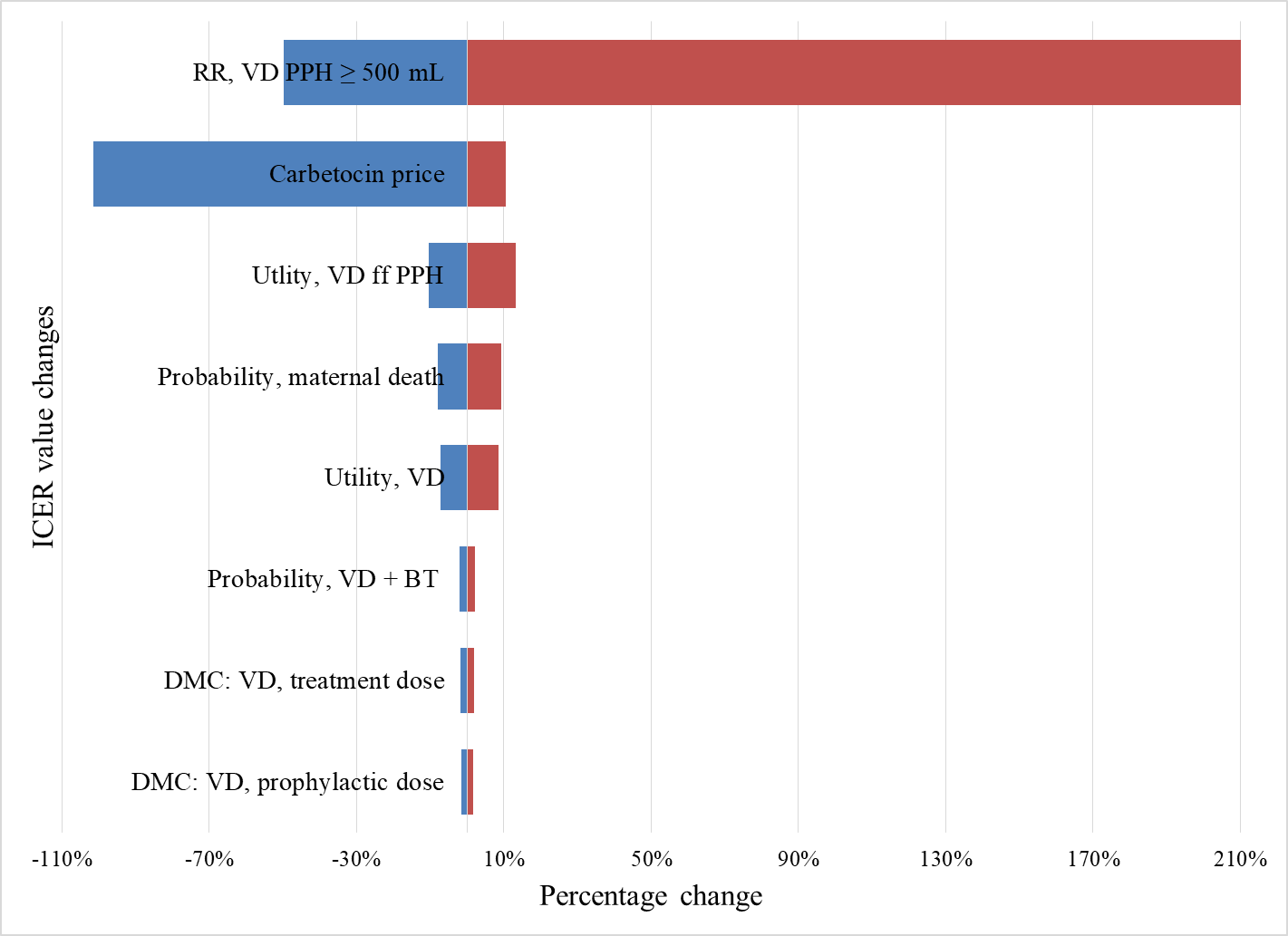


*RR-risk ratio, PPH-postpartum hemorrhage, Prob- probability, BT-blood transfusion, DMC- direct medical cost, IC-indirect cost*

Figure S1. Tornado diagram for parameters used in VD analysis


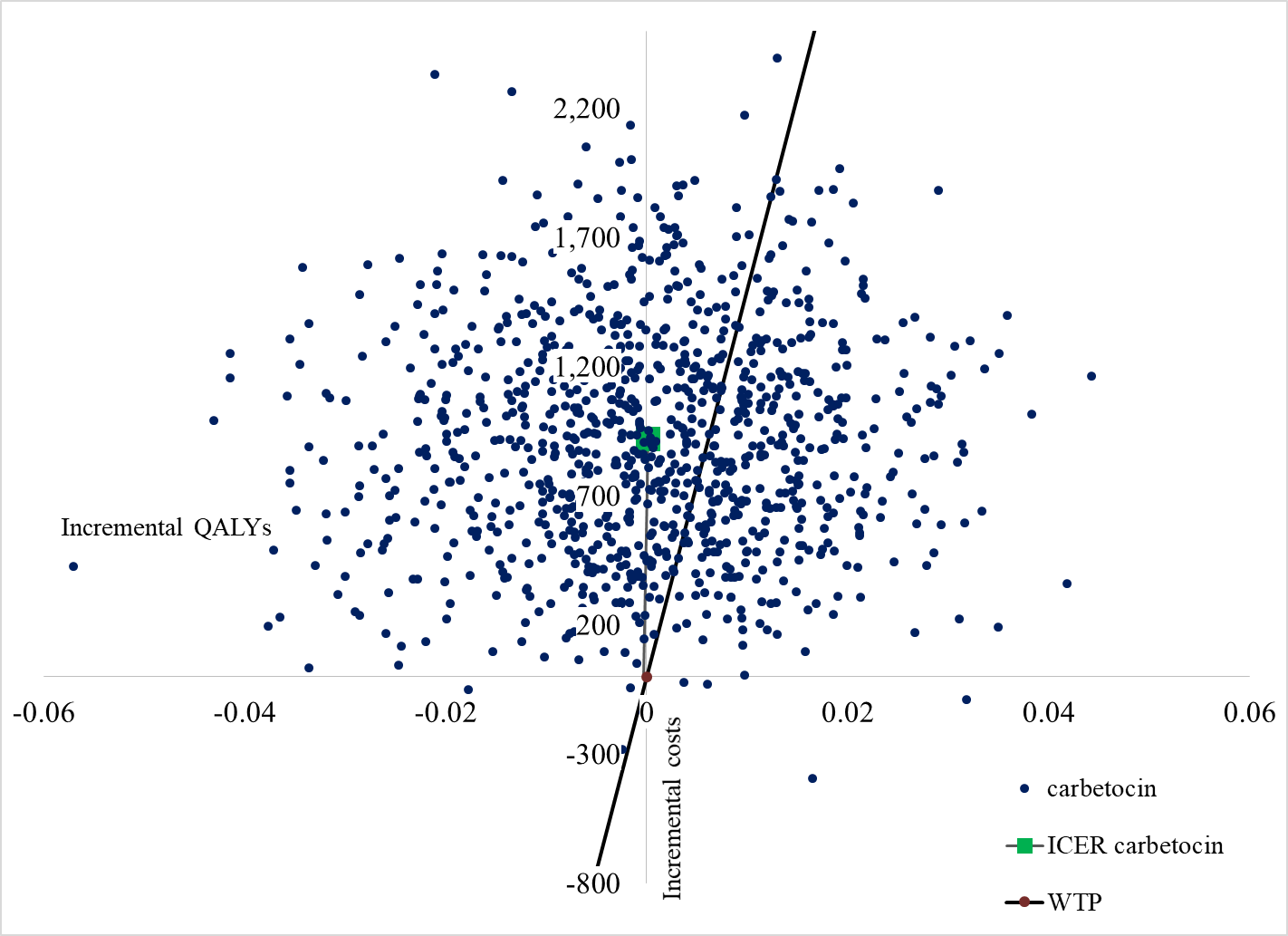


Figure S2. Cost-effectiveness plane of carbetocin compared with oxytocin for VD analysis


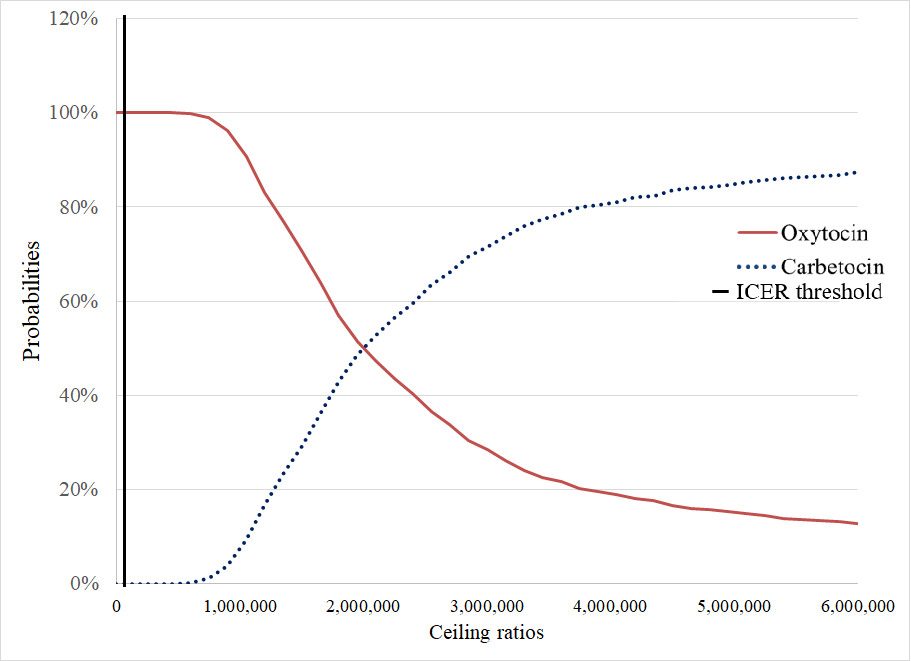


Figure S3. Cost-effectiveness acceptability curve for VD analysis

**Budget impact analysis**

This section shows the estimated budgetary requirements for the predicted product uptake versus 100% oxytocin use.

Table S3. Budget impact analysis results for cesarean section for 2020-2024 (*in millions*, USD)

| **Year** | **Drug mix ratio (Oxytocin: Carbetocin** | **Drug cost** | | **Delivery cost** | | **Product mix**  **(Oxytocin: Carbetocin)** | | **Total budget of product mix** | **Oxytocin**  **(100% uptake)** | | **Total budget of oxytocin**  **(100% uptake)** | **Budget impact** |
| --- | --- | --- | --- | --- | --- | --- | --- | --- | --- | --- | --- | --- |
|  |  | **Oxytocin** | **Carbetocin** | **Oxytocin** | **Carbetocin** | **Total drug cost** | **Total delivery cost** |  | **Total Drug cost** | **Total Delivery cost** |  |  |
| 2020 | 90:10 | 0.06 | 0.53 | 113.67 | 12.54 | 0.59 | 126.21 | 126.80 | 0.06 | 126.30 | 126.36 | 0.44 |
| 2021 | 85:15 | 0.05 | 0.79 | 106.28 | 18.62 | 0.84 | 124.90 | 125.74 | 0.06 | 125.04 | 125.10 | 0.64 |
| 2022 | 80:20 | 0.05 | 1.04 | 99.03 | 24.58 | 1.09 | 123.61 | 124.70 | 0.06 | 123.79 | 123.85 | 0.85 |
| 2023 | 75:25 | 0.05 | 1.28 | 91.91 | 30.42 | 1.33 | 122.33 | 123.66 | 0.06 | 122.55 | 122.61 | 1.05 |
| 2024 | 70:30 | 0.04 | 1.53 | 84.93 | 36.14 | 1.57 | 121.07 | 122.64 | 0.06 | 121.32 | 121.38 | 1.26 |
| **Total 5 year budget** | | **0.25** | **5.17** | **495.82** | **122.31** | **5.42** | **618.13** | **623.54** | **0.31** | **619.00** | **619.31** | **4.23** |

Table S4. Budget impact analysis results for vaginal delivery for 2020-2024 (*in millions*, USD)

| **Year** | **Drug mix ratio (Oxytocin: Carbetocin** | **Drug cost** | | **Delivery cost** | | **Product mix**  **(Oxytocin: Carbetocin)** | | **Total budget of product mix** | **Oxytocin**  **(100% uptake)** | | **Total budget of oxytocin**  **(100% uptake)** | **Budget impact** |  |
| --- | --- | --- | --- | --- | --- | --- | --- | --- | --- | --- | --- | --- | --- |
|  |  | **Oxytocin** | **Carbetocin** | **Oxytocin** | **Carbetocin** | **Total drug cost** | **Total delivery cost** |  | **Total Drug cost** | **Total Delivery cost** |  |  |  |
| 2020 | 90:10 | 0.24 | 2.24 | 184.57 | 20.48 | 2.48 | 205.05 | 207.53 | 0.27 | 205.08 | 205.35 | 2.18 |  |
| 2021 | 85:15 | 0.22 | 3.33 | 172.57 | 30.41 | 3.55 | 202.98 | 206.53 | 0.26 | 203.03 | 203.29 | 3.24 |  |
| 2022 | 80:20 | 0.21 | 4.40 | 160.80 | 40.14 | 4.61 | 200.94 | 205.55 | 0.26 | 201.00 | 201.26 | 4.29 |  |
| 2023 | 75:25 | 0.19 | 5.44 | 149.24 | 49.67 | 5.63 | 198.91 | 204.54 | 0.26 | 198.99 | 199.25 | 5.30 |  |
| 2024 | 70:30 | 0.18 | 6.47 | 137.90 | 59.01 | 6.65 | 196.91 | 203.56 | 0.26 | 197.00 | 197.26 | 6.30 |  |
| **Total 5 year budget** | | **1.04** | **21.88** | **805.08** | **199.71** | **22.92** | **1,004.79** | **1,027.71** | **1.31** | **1,005.09** | **1006.40** | **21.31** |  |

**References**

1. Lemeshow S HD, Klar J, Lwanga SK: . *Adequacy of sample size in health studies* Chichester: John Wiley and Sons 1990.

2. DiMaio H, Edwards RK, Euliano TY, et al. Vaginal birth after cesarean delivery: an historic cohort cost analysis. *Am J Obstet Gynecol* 2002; 186: 890-892. 2002/05/17.

3. Nigeen W, Farooq M, Afzal A, et al. Secondary postpartum haemorrhage in a tertiary care hospital of North India: a retrospective analysis. *International Journal of Reproduction, Contraception, Obstetrics and Gynecology* 2017; 6. DOI: 10.18203/2320-1770.ijrcog20170376.

4. Wright KN, Jonsdottir GM, Jorgensen S, et al. Costs and outcomes of abdominal, vaginal, laparoscopic and robotic hysterectomies. *JSLS : Journal of the Society of Laparoendoscopic Surgeons* 2012; 16: 519-524. 2013/03/15. DOI: 10.4293/108680812x13462882736736.

5. Kohler S, Sidney Annerstedt K, Diwan V, et al. Postpartum quality of life in Indian women after vaginal birth and cesarean section: a pilot study using the EQ-5D-5L descriptive system. *BMC Pregnancy Childbirth* 2018; 18: 427. 2018/10/31. DOI: 10.1186/s12884-018-2038-0.

6. Fennessy FM, Kong CY, Tempany CM, et al. Quality-of-life assessment of fibroid treatment options and outcomes. *Radiology* 2011; 259: 785-792. 2011/03/03. DOI: 10.1148/radiol.11100704.
